# Capsaicin Analogues Derived from *n*-3 Polyunsaturated Fatty Acids (PUFAs) Reduce Inflammatory Activity of Macrophages and Stimulate Insulin Secretion by β-Cells In Vitro

**DOI:** 10.3390/nu11040915

**Published:** 2019-04-24

**Authors:** Erika Cione, Pierluigi Plastina, Attilio Pingitore, Mariarita Perri, Maria Cristina Caroleo, Alessia Fazio, Renger Witkamp, Jocelijn Meijerink

**Affiliations:** 1 Department of Pharmacy, Health and Nutritional Sciences, Department of Excellence 2018-2022, University of Calabria, 87036 Arcavacata di Rende (CS), Italy; erika.cione@unical.it (E.C.); attiliopingitore@hotmail.com (A.P.); mariarita.perri@yahoo.it (M.P.); mariacristinacaroleo@virgilio.it (M.C.C.); alessia.fazio@unical.it (A.F.); 2 Division of Human Nutrition and Health, Wageningen University, 6700 AA Wageningen, The Netherlands; renger.witkamp@wur.nl (R.W.); jocelijn.meijerink@wur.nl (J.M.)

**Keywords:** diabetes, fatty acid amides, inflammation, obesity, PUFA, vanillylamide

## Abstract

In this study, two capsaicin analogues, *N*-eicosapentaenoyl vanillylamine (EPVA) and *N*-docosahexaenoyl vanillylamine (DHVA), were enzymatically synthesized from their corresponding *n*-3 long chain polyunsaturated fatty acids eicosapentaenoic acid (EPA) and docosahexaenoic acid (DHA), both dietary relevant components. The compounds significantly reduced the production of some lipopolysaccharide (LPS)-induced inflammatory mediators, including nitric oxide (NO), macrophage-inflammatory protein-3α (CCL20) and monocyte chemoattractant protein-1 (MCP-1 or CCL2), by RAW264.7 macrophages. Next to this, only EPVA increased insulin secretion by pancreatic INS-1 832/13 β-cells, while raising intracellular Ca^2+^ and ATP concentrations. This suggests that the stimulation of insulin release occurs through an increase in the intracellular ATP/ADP ratio in the first phase, while is calcium-mediated in the second phase. Although it is not yet known whether EPVA is endogenously produced, its potential therapeutic value for diabetes treatment merits further investigation.

## 1. Introduction

**Figure 1:**
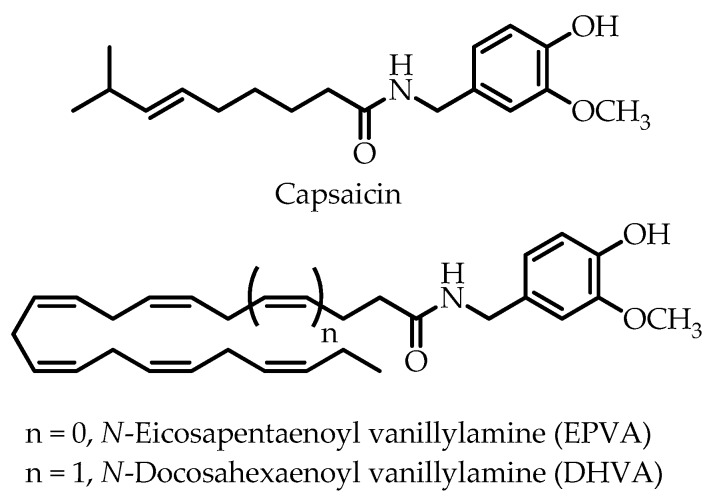
Chemical structures of capsaicin (CAP), *N*-eicosapentaenoyl vanillylamine (EPVA) and *N*-docosahexaenoyl vanillylamine (DHVA).

In the wake of the global obesity epidemic, the incidence of type 2 diabetes (T2D) continues to rise, creating major public health problems [1]. Type 2 diabetes is generally characterized by insufficient secretion of insulin from pancreatic β-cells, combined with impaired insulin sensitivity of peripheral tissues [2]. Impaired glucose-stimulated insulin secretion (GSIS) is a hallmark of β-cell dysfunction, which is commonly assumed to be secondary to the continued exposure to high glucose and lipid levels [3]. Type 2 diabetes is also associated with a chronically elevated inflammatory status which is typical for excess adipose tissue and endoplasmic reticulum stress. Several types of immune cells, including resident and recruited macrophages, become activated, thereby secreting various pro-inflammatory mediators including cytokines, chemokines and nitric oxide (NO), eventually contributing to β-cell dysfunction [4,5,6]. As a matter of fact, an increasing number of clinical studies show promising effects of anti-inflammatory drugs in T2D [7]. In view of this multifaceted etiology of T2D, compounds acting on multiple targets could provide helpful therapeutic additions. To this end, natural products, still often used in diabetes, could serve as lead structures, with metformin being a good example [4,8,9,10]. A product of interest is capsaicin (Figure 1), the major pungent component of red peppers [11,12], which has been reported to reduce blood glucose levels, increase insulin secretion, and elevate plasma insulin concentrations [13,14,15,16,17,18,19,20]. Next to this, studies demonstrated that capsaicin is primarily responsible for the anti-inflammatory properties of red pepper, acting by inhibiting the production and (or) secretion of pro-inflammatory mediators, including NO, prostaglandin E2 (PGE_2_), tumor necrosis factor (TNF)-α by macrophages [21,22,23,24,25,26]. Moreover, capsaicin was shown to inhibit mRNA expression of interleukin-6 (IL-6) and chemokine monocyte chemotactic protein-1 (MCP-1) and release of these mediators from adipose tissue and adipocytes of obese mice, whereas it enhanced adiponectin mRNA gene expression and protein release [27]. However, the use of capsaicin in clinical practice is limited by its side-effects, including hyperalgesia and intestinal complaints, like nausea and stomach cramps [28]. Structure activity relationship (SAR) studies showed that capsaicin analogues with longer acyl chains (R ≥ C16) and/or higher degree of unsaturation, such as *N*-palmitoyl vanillylamine (palvanil), *N*-oleoyl vanillylamine (olvanil) and *N*-arachidonoyl vanillylamine (arvanil), derived from palmitic, oleic and arachidonic acid, respectively, are non-pungent, and exhibit enhanced bioactivities and oral bioavailability compared to capsaicin [28,29,30,31,32,33]. Interestingly, the capsaicin analogue derived from the *n*-3 polyunsaturated fatty acid (PUFA) docosahexaenoic acid (DHA), *N*-docosahexaenoyl vanillylamine (called dohevanil by the authors), has been found to be more potent than capsaicin in inducing apoptosis in MCF-7 breast cancer cells [34]. The anti-inflammatory properties of *n*-3 PUFAs, including DHA and eicosapentaenoic acid (EPA) are well known [35,36]. Therefore, the purpose of the present study was to explore the potential anti-inflammatory properties in macrophages and the ability to modulate insulin secretion in pancreatic β-cells of *n*-3 polyunsaturated analogues of capsaicin, *N*-eicosapentaenoyl vanillylamine (EPVA) and *N*-docosahexaenoyl vanillylamine (DHVA) (Figure 1), enzymatically synthesized from their corresponding *n*-3 PUFA precursors.

## 2. Materials and Methods 

### 2.1. Chemicals and Materials

Vanillylamine, eicosapentaenoic acid (EPA), docosahexaenoic acid (DHA), triethylamine and 2-methyl-2-butanol were purchased from Sigma-Aldrich (Schnelldorf, Germany). Novozym^®^435 was from Novozymes (Bagsværd, Denmark). *n*-Hexane, acetone and methanol (analytical grade) were purchased from Carlo Erba Reagenti (Milan, Italy). Roswell Park Memorial Institute (RPMI)-1640, HEPES (4-(2-hydroxyethyl)-1-piperazineethanesulfonic acid), l-glutamine, sodium pyruvate, β-mercaptoethanol, and glucose were acquired from Thermofisher Scientific (Waltham, MA, USA). Dulbecco’s modified Eagle’s medium (DMEM), fetal bovine serum, streptomycin and penicillin were purchased from Lonza (Verviers, Belgium). LPS (*E. coli* O111:B4) was purchased from Sigma-Aldrich (Schnelldorf, Germany). Capsaicin, Griess reagents and nitrite standard were obtained from Cayman Chemical (Ann Arbor, MI, USA). The ELISA kit for determination of insulin was from Calbiotech Inc. (Spring Valley, CA, USA). The Ca^2+^ quantification kit was from Diagnosticum Rt (Budapest, Hungary). ATP was assayed with a bioluminescence kit from Thermofisher Scientific (Waltham, MA, USA). The ELISA kits for determination of MCP-1, and CCL20 were purchased from R&D Systems (Abingdon, UK).

### 2.2. Instrumentation

^1^H NMR and ^13^C NMR spectra were acquired at 25 °C on a Bruker AC 300 spectrometer in CDCl_3_ solutions at 300 MHz and 75 MHz, respectively, with tetramethylsilane (TMS) as the internal standard. Chemical shifts (*δ*) and coupling constants (*J*) are given in ppm and in Hz, respectively. IR spectra were acquired with a BIORAD FTS6000 spectrometer. Mass spectra were acquired using a Thermo LTQ XL Linear Ion Trap LC/MS mass spectrometer equipped with ion max source with electrospray ionization (ESI) probe in the positive or negative mode (ion spray voltage (IS) 5 kV (negative) or 4 kV (positive); capillary voltage −16 V (negative) or 15 V (positive); source temperature 300 °C) through direct infusion (5 μL min^−1^) of a methanolic solution of the compound (5 μg mL^−1^).

### 2.3. Synthesis and Characterization of N-Eicosapentaenoyl Vanillylamine (EPVA) and N-Docosahexaenoyl Vanillylamine (DHVA)

#### 2.3.1. Enzymatic Procedure

*N*-Eicosapentaenoyl vanillylamine (EPVA) and *N*-docosahexaenoyl vanillylamine (DHVA) were synthesized using our previously reported lipase-catalyzed *N*-acylation procedure as starting point [37,38,39] and adapted for the use of vanillylamine (Figure S1 in Supporting Materials). The appropriate fatty acid (eicosapentaenoic acid, EPA, 121 mg, or docosahexaenoic acid, DHA, 131 mg, 0.4 mmol), vanillylamine hydrochloride (76 mg, 0.4 mmol), and triethylamine (60 mg, 0.6 mmol) were let to react for 48 h in an orbital shaker at 50 °C using 2-methyl-2-butanol (2 mL) as the solvent, and Novozym^®^435 (consisting of immobilized *Candida antarctica* Lipase B, CALB) as the catalyst (100 mg). EPVA and DHVA were obtained after cooling, filtrating of the enzyme, and evaporating of the solvent under reduced pressure. Compounds’ purification was performed by column chromatography on silica gel (using *n*-hexane-acetone as the eluent) with a yield of 90 mg (51%) and 100 mg (54%), respectively. The purity was evaluated by HPLC and found to be ˃98% in both cases.

#### 2.3.2. Characterization Data

*N*-Eicosapentaenoyl vanillylamine (EPVA), colorless oil. IR (film) 3300 (s, br), 3011 (s), 2960 (m), 2930 (w), 1642 (s), 1515 (m) cm^−1^; ^1^H NMR δ 6.88–6.73 (m, 3 H on aromatic ring), 5.64 (s, br, 2 H, NH + OH), 5.45–5.28 (m, 10 H, 5 HC=CH), 4.35 (d, J = 5.5, 2 H, CH_2_NH), 3.87 (s, 3H, CH_3_O), 2.87–2.75 (m, 8 H, 4 CH=CHCH_2_CH=CH), 2.24–2.17 (m, 2 H, CH_2_C=O), 2.14–2.04 (m, 4 H, CH_2_CH_2_CH_2_C=O + CH_2_CH_3_), 1.80–1.69 (m, 2 H, CH_2_CH_2_C=O), 0.97 (t, J = 7.6, 3 H, CH_2_CH_3_); ^13^C NMR δ 171.4, 146.1, 144.7, 131.2, 129.8, 128.3, 128.0, 127.8, 127.5, 127.4, 127.3, 127.1, 126.3, 120.1, 113.8, 110.3, 55.5, 42.8, 35.3, 28.7, 25.9, 24.8, 24.7, 21.7, 19.6, 13.1; MS (ESI+, direct infusion) m/z = 438 (100% [M + H]^+^). MS/MS [M + H]^+^ (ESI+, 15 eV): m/z = 438.37 (31), 302.25 (100). MS (ESI−, direct infusion) m/z = 436 (100% [M − H]^−^). MS/MS [M − H]^−^ (ESI−, 15 eV): m/z = 436.20 (19), 300.32 (100).

*N*-Docosahexaenoyl vanillylamine (DHVA), colorless oil. IR (film) 3300 (s, br), 3016 (s), 2965 (m), 2935 (w), 1647 (s), 1515 (m) cm^−1^; ^1^H NMR δ 6.88–6.73 (m, 3H on aromatic ring), 5.66 (s, br, 2H, NH + OH), 5.50–5.30 (m, 12 H, 6 HC=CH), 4.35 (d, J = 5.7, 2H, CH_2_NH), 3.88 (s, 3H, CH_3_O), 2.92−2.78 (m, 10H, 5CH=CHCH_2_CH=CH), 2.46−2.40 (m, 2H, CH_2_C=O), 2.30−2.25 (m, 2H, CH_2_CH_2_C=O), 2.12−2.04 (m, 2 H, CH_2_CH_3_), 0.97 (t, J = 7.5, 3 H, CH_2_CH_3_); ^13^C NMR δ 172.0, 146.7, 145.2, 132.1, 130.3, 129.4, 128.6, 128.4, 128.34, 128.31, 128.2, 128.1, 128.0, 127.9, 127.1, 120.9, 114.4, 110.8, 56.0, 43.6, 36.5, 29.7, 25.7, 25.6, 23.5, 20.6, 14.3; MS (ESI+, direct infusion) m/z = 464 (100% [M + H]^+^). MS/MS [M + H]^+^ (ESI+, 12 eV): m/z = 464.40 (29), 328.27 (100). MS (ESI−, direct infusion) m/z = 462 (100% [M − H]^−^). MS/MS [M − H]^−^ (ESI−, 12 eV): m/z = 462.40 (31), 326.37 (100).

### 2.4. Cell Culture

#### 2.4.1. RAW 264.7 Macrophages

Murine RAW264.7 macrophages were acquired from the American Type Culture Collection (Teddington, UK). Dulbecco’s modified Eagle’s medium (DMEM) supplemented with 10% fetal bovine serum, streptomycin and penicillin was used to grow and maintain cells at 37 °C in a 5% CO_2_ humidified air atmosphere.

#### 2.4.2. Pancreatic β-Cells

Pancreatic INS-1 832/13 β-cell line (provided by Prof. C. Newgard, Duke University, Durham, NC, USA) was used. Insulin secreting INS-1 832/13 cells, stably transfected with a plasmid coding for human Proinsulin [40], were maintained in RPMI-1640 medium supplemented with 10% fetal bovine serum, 10 mM HEPES, 2 mM l-glutamine, 1 mM sodium pyruvate, 50 mM β-mercaptoethanol, 100 IU mL^−1^ penicillin, and 100 IU mL^−1^ streptomycin. Cells were incubated under 95% O_2_, 5% CO_2_ at 37 °C. Media were refreshed every 2–3 days and cells were trypsinized and passaged weekly. Cells were subcultured when they approached ≥70% confluence.

### 2.5. Viability and Cytotoxicity Assays

Cell viability and potential cytotoxicity effects in RAW264.7 cells were assessed by measuring tetrazolium salt (XTT) conversion and lactate dehydrogenase (LDH) leakage, respectively, as previously reported [41]. For XTT conversion, XTT Cell Proliferation Kit II (Roche Applied Science, Almere, The Netherlands) was used. Briefly, after 48 h incubation of RAW264.7 cells with the compounds and LPS, supernatants were removed (and used for LDH determination) and fresh medium (100 μL) containing sodium 30-[1-(phenylaminocarbonyl)-3,4-tetrazolium]bis(4-methoxy-6-nitro) benzenesulfonic acid hydrate (XTT) (final concentration = 0.45 mM) and *N*-methyldibenzopyrazine methylsulfate (1.25 mM), was added to the cells. After incubating at 37 °C, the quantity of formazan formed in the medium was evaluated at 450 nm on a plate reader (Multiskan Ascent, ThermoLabsystem, Breda, The Netherlands). LDH leakage was measured using a Cytotoxicity Detection Kit (Roche Applied Science, Almere, The Netherlands). LDH was evaluated in culture supernatants (100 μL), previously taken and mixed with enzyme reagents (diaphorase/NAD mixture, 250 μL) and dye solutions (iodotetrazolium chloride and sodium lactate, 11.25 mL). After incubating for 30 min at 25 °C, the absorbance was measured at 492 nm.

Pancreatic INS-1 832/13 β-cell line was seeded in 96-well plates. After overnight attachment, cells were incubated for 24 h with test compounds at the final concentration of 2.5 µM, in duplicate. DMSO was used as negative control. Then, MTT [(3-(4,5-dimethylthiazol-2-yl)-2,5-diphenyltetrazolium bromide)] solution in DPBS (3 mg mL^−1^) was added and cells were incubated for another 6 h. Mitochondrial reductase enzymes in viable cells reduce the yellow tetrazolium MTT in its formazan, which has a purple color when dissolved in DMSO under basic condition [42,43]. Media were finally replaced with 100 µL of DMSO and cell viability was evaluated by measuring absorbance of the colored wells at 545 nm, using Multi-Mode Microplate Reader (Synergy H1-BioTeck, Winooski, VT, USA).

### 2.6. Nitric Oxide Quantification

Cells were seeded into 96-well cell culture plates (2.5 × 10^5^ cells mL^−1^), and incubated overnight. Adherent cells were incubated with (or without) LPS (0.5 mg mL^−1^) in the presence (or absence, containing vehicle only) of the test compounds for 48 h. Nitrite accumulated in macrophage culture medium, after incubation, was measured as an estimation of NO amount, as described previously [39]. Briefly, cell culture media (100 µL) were let to react with Griess reagents (100 µL) and incubated at room temperature for 10 min. Absorbance was measured at 540 nm using an ELISA plate reader.

### 2.7. Effects of EPVA and DHVA on MCP-1, CCL20 and IL-6 Production

Cells were seeded into 48-well plates (5 × 10^5^ cells mL^−1^). After overnight incubation, adherent cells were treated with (or without) LPS (0.5 mg mL^−1^) in the presence (or absence, containing vehicle only) of the test compounds for 24 h. Protein levels were measured by using ELISA in agreement with the manufacturer’s instructions.

### 2.8. Quantification of Insulin Secretion

Pancreatic INS-1 832/13 β-cells (5 × 10^5^ cells mL^−1^) were plated in 24-well plates with RPMI-1640, 11 mM Glucose, 10% FBS. The next day, the medium was switched to 5 mM Glucose, 10 % FBS. After 16 h of incubation, β-cells were washed and secretion media (Hanks’ balanced salts, HBSS with 20 mM Hepes and 1% BSA, pH 7.2) containing 3 mM glucose were added. After 2 h, secretion media were replaced by fresh secretion media (GSIS) containing 23 mM glucose, with (or without, containing vehicle only) test compounds (2.5 µM) for 15 or 120 min of incubation. Amount of insulin in cell medium was analyzed by insulin ELISA kit (Calbiotech Inc.) in agreement with the manufacturer’s instructions. Briefly, the medium was spun 5 min at 2500 rpm, 4 °C, to pellet down cellular debris and either immediately assayed or frozen in liquid N_2_ first and −80 °C successively, for no longer than one week. 

### 2.9. β-Cell Line Function Assays

To assay Ca^2+^ and ATP content, β-cells were transferred to 12-well plates till reaching 85% of confluence. Medium containing 5 mM glucose was replaced by growth medium for 16 h. Before assaying, this medium was replaced by 2 mL HBSS containing 3 mM glucose for 2 h. Experiments were then started by replacing the HBSS to 0.8 mL per well of either fresh secretion buffer with 23 mM glucose, with (or without, containing vehicle only) test compounds (2.5 µM) for 15 or 120 min of incubation. Ca^2+^ was measured by a kit in agreement with the manufacturer’s instructions (Diagnosticum Rt) in cells that were added with 0.1 mL of radioimmunoprecipitation assay (RIPA) buffer, scraped several times, and centrifuged to remove cellular debris (10 min, 4 °C). ATP was measured by a bioluminescence kit (ATP determination kit; Molecular Probes, Eugene, OR, USA) in cells treated with reporter lysis buffer (Promega, Madison, WI, USA) containing a protease inhibitor mixture (Sigma, Schnelldorf, Germany). An aliquot of each sample was let to react with 100 μL of 20 × reaction buffer, 1 M DTT, containing firefly luciferase and 10 mM luciferin. Emitted light was measured in a luminometer (Berthold, Bad Wildbad, Germany).

### 2.10. Statistical Analysis

All experiments were performed in duplicate in at least three independent experiments. Data are presented as means ± standard deviation. Data from experiments with RAW264.7 macrophages are shown as percentage of the LPS-treated controls (set at 100%). Statistical differences between treatments and controls were assessed by one-way ANOVA followed by Dunnett’s post hoc test. Statistically significant differences were considered for *p* < 0.05 (*), *p* < 0.01 (**), and *p* < 0.001 (***).

## 3. Results

### 3.1. Effect of N-Eicosapentaenoyl Vanillylamine (EPVA) and N-Docosahexaenoyl Vanillylamine (DHVA) on the Production of NO

**Figure 2:**
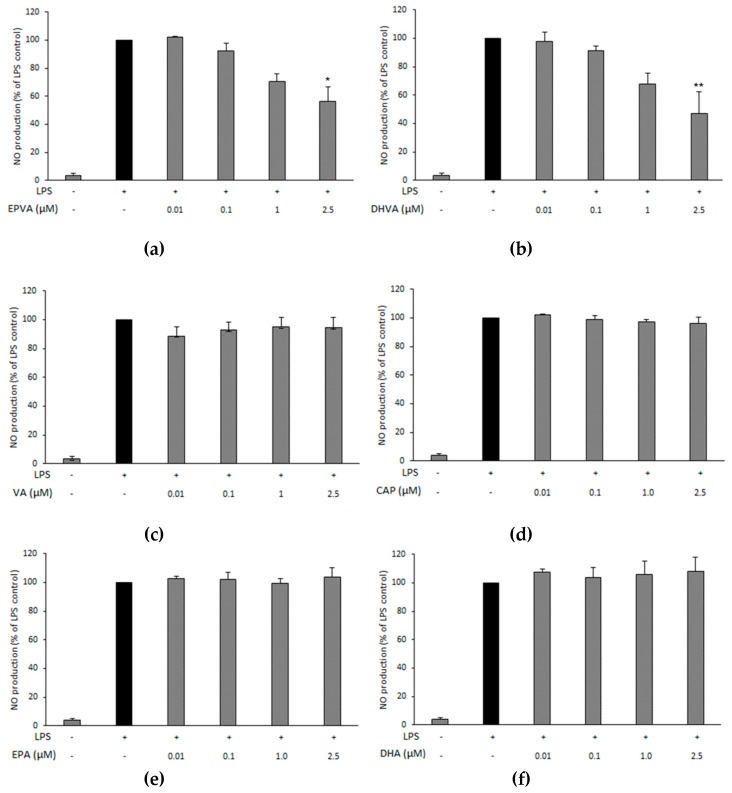
Effect of *N*-eicosapentaenoyl vanillylamine (EPVA, panel **a**), *N*-docosahexaenoyl vanillylamine (DHVA, **b**), vanillylamine (VA, **c**), capsaicin (CAP, **d**), eicosapentaenoic acid (EPA, **e**) and docosahexaenoic acid (DHA, **f**) on the production of nitric oxide (NO) by lipopolysaccharide (LPS)-stimulated RAW264.7 macrophages. Cells were seeded in 96-well plates at 2.5 × 10^5^ cells mL^−1^. After overnight incubation, they were added of LPS (0.5 μg mL^−1^) and compounds (or vehicle) at different concentrations for 48 h. Cell supernatants were examined for nitrite production by Griess assay. Data are shown as percentage, and LPS control (without compounds) was set at 100%, and represents the mean of three separate experiments (each done in duplicate) ± standard deviation. Mean value was statistically different from control (* *p* < 0.05, and ** *p* < 0.01).

The potential anti-inflammatory properties of EPVA and DHVA (in a concentration range 10 nM–2.5 μM) were investigated by RAW264.7 macrophages induced with 0.5 μg mL^−1^ of LPS and incubated for 48 h. Both EPVA and DHVA inhibited NO release in a significant way at their highest concentration (2.5 μM) up to 56% and 47%, respectively (Figure 2a,b). Neither EPVA nor DHVA showed effects on cell viability or cytotoxicity in the concentration ranges tested (Table S1 in Supporting Information). Vanillylamine (VA), capsaicin (CAP) and the fatty acid precursors, EPA and DHA, did not influence the production of NO (Figure 2c–f).

### 3.2. Effect of N-Eicosapentaenoyl Vanillylamine (EPVA) and N-Docosahexaenoyl Vanillylamine (DHVA) on the Production of MCP-1, CCL20 and IL-6

**Figure 3:**
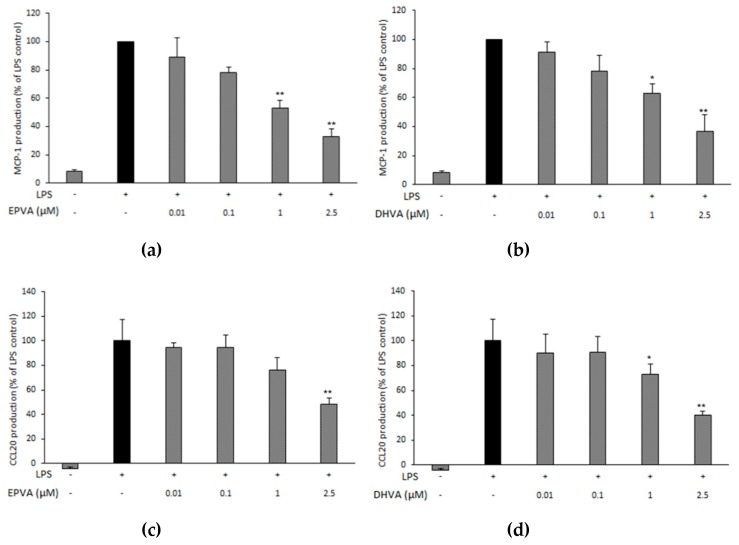
Effect of *N*-eicosapentaenoyl vanillylamine (EPVA) and *N*-docosahexaenoyl vanillylamine (DHVA) on the production of the chemokine monocyte chemoattractant protein-1 (MCP-1) (panels **a** and **b**) and macrophage-inflammatory protein-3α (CCL20) (panels **c** and **d**), by RAW264.7 macrophages stimulated with lipopolysaccharide (LPS). Cells were plated in 48-wells at 5 × 10^5^ cells mL^−1^. After overnight incubation, they were added of LPS (0.5 μg mL^−1^) and compounds (or vehicle) at different concentrations for 24 h. Cell supernatants were analyzed for protein levels using ELISA. Data are shown as percentage, where LPS control (without compounds) was set at 100%, and represents the mean of three distinct experiments (each performed in duplicate) ± standard deviation. Mean value was statistically different from control (* *p* < 0.05, and ** *p* < 0.01).

We further investigated the potential of EPVA and DHVA on the production of other pro-inflammatory markers, namely the chemokine monocyte chemotactic protein-1 (MCP-1 or CCL2), the macrophage-inflammatory protein-3α (CCL-20), and interleukin-6 (IL-6), by RAW264.7 cells stimulated with 0.5 μg mL^−1^ of LPS and incubated for 24 h. Both compounds reduced MCP-1 production in a concentration-dependent way (Figure 3a,b). In particular, EPVA and DHVA caused 67% and 64% reduction at their highest concentration (2.5 μM), respectively, and were also effective in reducing MCP-1 production at 1 μM. Both compounds were also able to reduce CCL20 production at the highest concentration (2.5 μM), with DHVA being already effective at 1 μM (Figure 3c,d). By contrast, EPVA and DHVA were ineffective in reducing IL-6 production at all concentrations tested.

### 3.3. Effect of N-Eicosapentaenoyl Vanillylamine (EPVA) and N-Docosahexaenoyl Vanillylamine (DHVA) on Insulin Secretion

**Figure 4:**
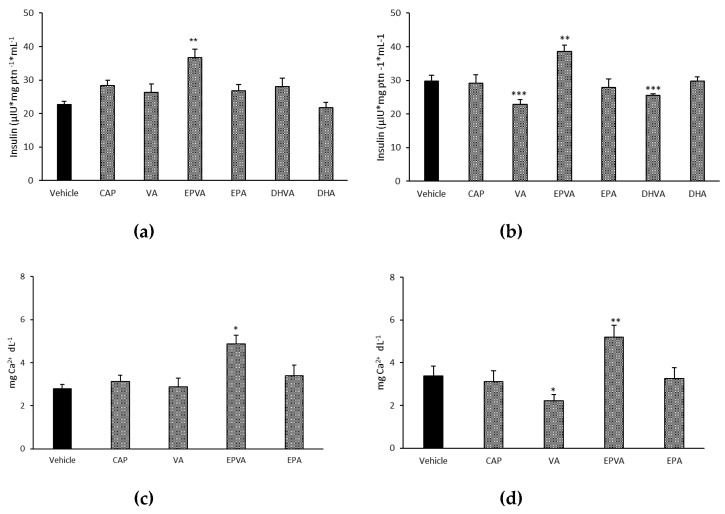
Effects of capsaicin (CAP), vanillylamine (VA), *N*-eicosapentaenoyl vanillylamine (EPVA), eicosapentaenoic acid (EPA), *N*-docosahexaenoyl vanillylamine (DHVA) and docosahexaenoic acid (DHA) at the concentration of 2.5 µM on insulin secretion in pancreatic β-cells INS-1 832/13 after 15 min (**a**), and after 120 min (**b**). Effects of capsaicin (CAP), vanillylamine (VA), *N*-eicosapentaenoyl vanillylamine (EPVA) and eicosapentaenoic acid (EPA) at the concentration of 2.5 µM on Ca^2+^ amounts in pancreatic β-cells INS-1 832/13 after 15 min (**c**), and after 120 min (**d**). Mean value was statistically different from control (* *p* < 0.05, ** *p* < 0.01, and *** *p* < 0.001).

The effects of EPVA and DHVA (at the concentration of 2.5 µM) on glucose-induced insulin secretion were investigated in pancreatic β-cells INS-1 832/13, at both short and long incubation time. No toxicity was observed upon treatment with the compounds (Table S2 in Supporting Information). EPVA induced a significant increase in the release of insulin by almost 42% (Figure 4a) compared to control after 15 min of incubation, whereas DHVA was ineffective (Figure 4a). After 120 min, EPVA kept insulin secretion levels higher compared to the control, while DHVA induced a slight but significant decrease of insulin secretion (Figure 4b). Remarkably, capsaicin (CAP) was ineffective at both incubation times. We also tested the effect of vanillylamine (VA) and the precursors of EPVA and DHVA, EPA and DHA, respectively, to rule out the possibility that the observed effects were produced by the corresponding hydrolysis products. Neither EPA nor DHA had any effects at both short and long time, while VA induced a decrease (by 23%) of insulin secretion at 120 min of incubation (Figure 4b).

### 3.4. Effect of N-Eicosapentaenoyl Vanillylamine (EPVA) and N-Docosahexaenoyl Vanillylamine (DHVA) on Intracellular ATP Levels

A crucial event in glucose-induced insulin release is the induction of mitochondrial oxidative metabolism. During GSIS condition, enhanced ATP synthesis leads to the closure of ATP-sensitive K^+^ (KATP) channels followed by membrane depolarization and Ca^2+^ influx via voltage-gated Ca^2+^ channels, which in turn triggers release of insulin [44]. Therefore, we also tested the effect of the hybrid compounds as well as their precursors (at the concentration of 2.5 µM) on ATP production in INS-1 832/13 cell line. ATP amount in β-cells was found to be increased only following 15 min of incubation with 2.5 µM EPVA, whereas after 120 min of incubation ATP amount did not differ from the control (Table 1). None of the other compounds had the ability to change the ATP levels in pancreatic β-cells at both time points investigated.

### 3.5. Effect of N-Eicosapentaenoyl Vanillylamine (EPVA) and N-Docosahexaenoyl Vanillylamine (DHVA) on Intracellular Ca^2+^ Levels

Since EPVA was able to stimulate insulin release and ATP production, Ca^2+^ levels were also measured. The amount of intracellular Ca^2+^ was significantly increased (by 74%) following EPVA treatment both after 15 min (Figure 4c), and 120 min of incubation (Figure 4d). Capsaicin and EPA were ineffective at both time points, while VA induced a significant decrease of Ca^2+^ amount by 35% after 120 min of incubation (Figure 4d).

## 4. Discussion

Fatty acid amides are conjugates of fatty acids with amines, including ethanolamine, dopamine and serotonin, or amino acids. These compounds are abundantly present in nature and display a variety of bioactivities [45]. Fatty acid amides derived from *n*-3 polyunsaturated fatty acids (PUFAs) represent a sub-class which is only quite recently receiving attention [46]. Among them, *N*-eicosapentaenoyl ethanolamine (EPEA) and *N*-docosahexaenoyl ethanolamine (DHEA) were shown to possess antitumor activity in prostate and breast cancer cell lines [47,48] and anti-inflammatory properties in adipocytes and macrophages [49,50,51]. Another congener, *N*-docosahexaenoyl dopamine (DHDA), displayed antitumor activity in breast cancer cells [38] and anti-inflammatory properties in macrophages and microglial cells [39], while *N*-docosahexaenoyl serotonin (DHA-5-HT) attenuated the production of pro-inflammatory markers in macrophages and human peripheral blood mononuclear cells [52,53]. Collectively, studies indicate that amides from *n*-3 PUFAs display more potent anti-inflammatory activities than compounds bearing shorter or saturated chains [51,53,54]. Besides, data indicate that the structure of the conjugated amine is also important for bioactivity. For example, from a series of structural analogues tested, dopamine conjugates were found to be the most active in reducing inflammatory markers [54]. Vanillyl conjugates have been studied mainly in comparison with capsaicin. In this respect, palvanil has been reported to inhibit inflammatory and neuropatic pain [31], while olvanil, and alvanil were found to display anti-invasive activity in human small cell lung cancers [28]. Thus far, vanillyl conjugates with *n*-3 PUFAs have received little attention, with the exception of *N*-docosahexaenoyl vanillylamine (DHVA) which was found to induce apoptosis in MCF-7 breast cancer cells [34]. 

The results from the present work show that, at the concentration of 2.5 µM, vanillyl conjugates from EPA and DHA decreased NO production by LPS-induced RAW264.7 cells. Additionally, we observed that EPVA reduced MCP-1 production in a concentration-dependent manner and CCL20 release at the highest concentration tested in LPS-induced cells. Parallel to this, DHVA caused a concentration-dependent decrease of both MCP-1 and CCL20. Nitric oxide is a late marker of the LPS inflammatory cascade and originates from the oxidative deamination of l-arginine, which is catalyzed by the inducible isoform of nitric oxide synthase (iNOS) [55]. Mice in which iNOS has been knocked out do not develop insulin resistance when fed with a high fat diet, unlike their wild type (WT) mice littermates [56,57]. MCP-1 and CCL20 are two chemokines playing important roles in attracting immune cells to inflammation site, thus inducing the amplification of the inflammatory reaction [58,59]. MCP-1 is known to be one of the key players in adipose tissue inflammation during obesity and its associated metabolic complications [60]. CCL20 is assumed to contribute to the development and progression of obesity-associated insulin resistance, during which its levels were found to be raised in adipose tissue, plasma as well as in pancreatic β-cells [61]. The association between inflammation and T2D and the idea that anti-inflammatory compounds might be potential antidiabetic leads [4,5,6], prompted us to investigate the ability of EPVA and DHVA to modulate insulin secretion from pancreatic β-cells. Using hybrid molecules from food-derived compounds has been suggested as a new approach for insulin secretion studies [62]. The INS-1 832/13 cell line represents the most physiologically relevant in vitro β-cell model currently available and often used to screen and study biochemical mechanism of endogenous/exogenous molecules [63,64]. We show that EPVA increased insulin secretion, while capsaicin was not effective at the concentration investigated. Parallel to this, we found that EPVA increased the amounts of intracellular Ca^2+^ and ATP, suggesting that the mechanism of insulin release is mediated via an increase of the intracellular ATP/ADP ratio with the consequent increase of Ca^2+^ influx through the voltage-gated channels. The higher activity of EPVA compared to capsaicin could be attributed to its longer chain and higher degree of unsaturation. These features have been reported to play a role in the bioactivities, such as anti-inflammatory and anticancer properties, of other long chain analogues of capsaicin [28,31,34]. At the same time, this argument would seem to be in contrast with the observation that DHVA, having an even higher chain, did not show much activity in this assay. In contrast to EPVA, we did not observe a significant increase in intracellular calcium with EPA treatment. This underlines that the insulin secretory effect of EPVA is not due to its breakdown into EPA and vanillylamine, as both compounds displayed no effect on insulin secretion at the concentration investigated in our study. Interestingly, in mice co-administration of capsaicin and EPA has been recently shown to lead to beneficial effects on delaying the progression of obesity-related metabolic dysregulation and, therefore, its complications [65]. In this animal study, improvement of insulin resistance, indicated by the HOMA-IR index, was associated with decreased serum glucose and enhanced serum insulin levels in the EPA- and capsaicin-treated mice group. Authors attributed this effect to the decrease in adipose tissue weight that indirectly affects insulin secretion. Herein we showed that EPVA is able to increase insulin secretion from pancreatic β-cells in vitro. Although we have no evidence yet for the existence of EPVA or DHVA as endogenous compounds, it is worth highlighting that we and others have demonstrated the formation of *n*-3 PUFA-derived amides including DHA-5-HT and DHEA in human and other species. Their biosynthesis was found to be tissue-specific and influenced by dietary content [49,53]. Therefore, we could speculate that EPVA and DHVA might be endogenously formed when consuming a diet rich in capsaicin and *n*-3 PUFAs.

## 5. Conclusions

In conclusion, two capsaicin analogues EPVA and DHVA were enzymatically synthesized from their corresponding *n*-3 PUFAs. Both compounds significantly reduced the production of some inflammatory mediators, including NO, CCL20 and MCP-1, by LPS-stimulated RAW264.7 macrophages. Next to this, EPVA increased insulin secretion by pancreatic INS-1 832/13 β-cells, while raising intracellular Ca^2+^ and ATP concentrations. This merits further investigation into its possible endogenous role and (or) its therapeutic potential in diabetes.

## Figures and Tables

**Table 1:** Measurement of ATP levels in INS-1 832/13 cells.

Compound	ATP Concentration (nM) ^1^		
	0 min	15 min	120 min
Vehicle	2.80 ± 0.71	4.65 ± 0.23	4.78 ± 0.72
Capsaicin (CAP)	1.99 ± 0.55	5.24 ± 0.85	4.12 ± 0.37
Vanillylamine (VA)	2.56 ± 0.99	4.31 ± 0.52	3.12 ± 0.21
Eicosapentaenoic acid (EPA)	2.39 ± 0.68	5.88 ± 0.32	5.24 ± 0.62
Docosahexaenoic acid (DHA)	2.01 ± 0.91	3.98 ± 0.93	4.69 ± 0.69
*N*-Eicosapentaenoyl vanillylamine (EPVA)	2.19 ± 0.81	12.56 ± 0.57 ***	4.59 ± 0.55
*N*-docosahexaenoyl vanillylamine (DHVA)	2.46 ± 0.82	4.56 ± 0.56	3.64 ± 0.81

^1^ Cells were treated with compounds (at the concentration of 2.5 µM) or DMSO (as vehicle) and ATP at 0 (baseline), 15 and 120 min was quantified as described in Materials and methods. Data are presented as mean ± SD of triplicate experiments. Mean value was statistically different from vehicle control at 15 min (*** *p* < 0.001).

## Supplementary Materials

The following are available online at https://www.mdpi.com/2072-6643/11/4/915/s1, Figure S1: Synthesis of *N*-eicosapentaenoyl vanillylamine (EPVA) and *N*-docosahexaenoyl vanillylamine (DHVA), Table S1: Effect of EPVA and DHVA on RAW 264.7 macrophages by XTT / LDH assays, Table S2: Effect of EPVA and DHVA on INS-1 832/13 β-cell line by MTT assay.
