# Supplementary material for: Capsaicin Analogues Derived from n-3 Polyunsaturated Fatty Acids (PUFAs) Reduce Inflammatory Activity of Macrophages and Stimulate Insulin Secretion by β-Cells In Vitro
Source: Nutrients. 2019 Apr 24;11(4):915. doi: 10.3390/nu11040915 (PMC6520993; doi:10.3390/nu11040915)
Supplement: Supplementary file 1 [file nutrients-11-00915-s001.pdf]

Capsaicin analogues derived from n-3 polyunsaturated fatty acids (PUFAs) reduce inflammatory activity of macrophages and stimulate insulin secretion by β-cells *in vitro*

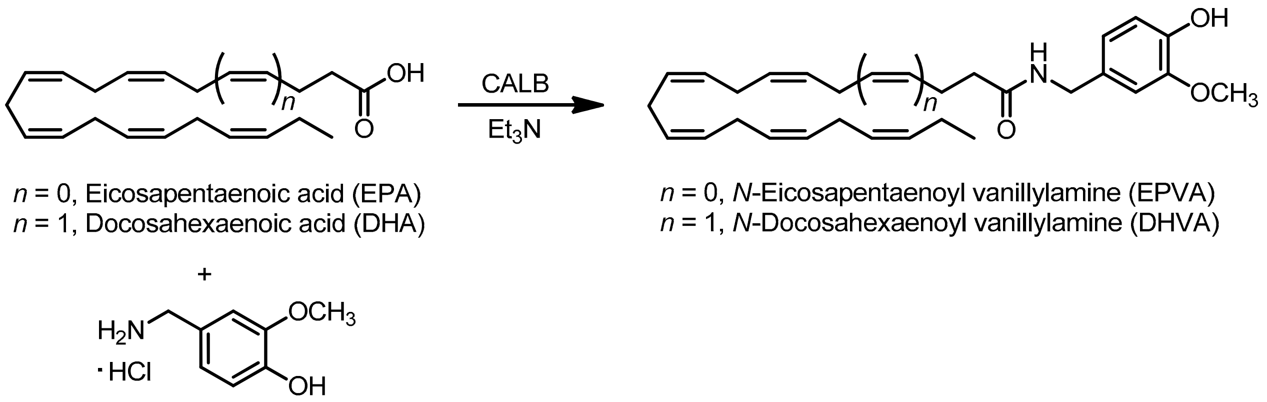

Figure S1. Synthesis of *N*-eicosapentaenoyl vanillylamine (EPVA) and *N*-docosahexaenoyl vanillylamine (DHVA)

Table S1: Effect of EPVA and DHVA on RAW 264.7 macrophages by XTT / LDH assays

Treatment	Concentration (µM)	Viability (%)	Cytoxicity (%)
Vehicle	-	100	100
EPVA	0.01	116 ± 3	100 ± 4
	0.1	118 ± 12	105 ± 4
	1.0	99 ± 7	100 ± 5
	2.5	109 ± 12	112 ± 9
DHVA	0.01	119 ± 19	103 ± 2
	0.1	97 ± 16	100 ± 1
	1.0	108 ± 11	103 ± 9
	2.5	107 ± 8	111 ± 6
Triton X-100	-	27 ± 2	212 ± 6

Table S2: Effect of EPVA and DHVA on INS-1 832/13 β-cell line by MTT assay

Treatment	Concentration (µM)	Viability (%)
Vehicle	-	100
EPVA	1.0	99 ± 6
	2.5	95 ± 7
DHVA	1.0	89 ± 13
	2.5	83 ± 11
